# Do Australian general practitioners believe practice nurses can take a role in chlamydia testing? A qualitative study of attitudes and opinions

**DOI:** 10.1186/s12879-015-0757-7

**Published:** 2015-01-31

**Authors:** Rebecca Lorch, Jane Hocking, Rebecca Guy, Alaina Vaisey, Anna Wood, Basil Donovan, Christopher Fairley, Jane Gunn, John Kaldor, Meredith Temple-Smith, on behalf of the ACCEPt consortium

**Affiliations:** The Kirby Institute for Infection and Immunity in Society, UNSW Australia, Sydney, NSW Australia; Centre for Epidemiology and Biostatistics, Melbourne School of Population and Global Health, University of Melbourne, Melbourne, Victoria Australia; Sydney Sexual Health Centre, Sydney Hospital, Sydney, NSW Australia; Central Clinical School, Monash University and Melbourne Sexual Health Centre, Carlton, Victoria Australia; Department of General Practice, University of Melbourne, Melbourne, Victoria Australia

## Abstract

**Background:**

Chlamydia notifications continue to rise in young people in many countries and regular chlamydia testing is an important prevention strategy. Although there have been initiatives to increase testing in primary care, none have specifically investigated the role of practice nurses (PNs) in maximising testing rates. PNs have previously expressed a willingness to be involved, but noted lack of support from general practitioners (GPs) as a barrier. We sought GPs’ attitudes and opinions on PNs taking an expanded role in chlamydia testing and partner notification.

**Methods:**

In the context of a cluster randomised trial in mostly rural towns in 4 Australian states, semi structured interviews were conducted with 44 GPs between March 2011 and July 2012. Data relating to PN involvement in chlamydia testing were thematically analysed using a conventional content analysis approach.

**Results:**

The majority of GPs interviewed felt that a role for PNs in chlamydia testing was appropriate. GPs felt that PNs had more time for patient education and advice, that patients would find PNs easier to talk to and less intimidating than GPs, and that GPs themselves could benefit through a reduction in their workload. Although GPs felt that PNs could be utilised more effectively for preventative health activities such as chlamydia testing, many raised concerns about how these activities would be renumerated whilst some felt that existing workload pressures for PNs could make it difficult for them to expand their role. Whilst some rural GPs recognised that PNs might be well placed to conduct partner notification, they also recognised that issues of patient privacy and confidentiality related to living in a “small town” was also a concern.

**Conclusion:**

This is the first qualitative study to explore GPs’ views around an increased role for PNs in chlamydia testing. Despite the concerns raised by PNs, these findings suggest that GPs support the concept and recognise that PNs are suited to the role. However issues raised, such as funding and remuneration may act as barriers that will need to be addressed before PNs are supported to make a contribution to increasing chlamydia testing rates in general practice.

## Background

Throughout the western world, *Chlamydia trachomatis* infection (hereafter referred to as chlamydia) continues to be of significant public health concern. Affecting mainly young adults aged 15–29 years, chlamydia is the most commonly diagnosed bacterial sexually transmitted infection in the United States, Europe and also Australia, where over 82 000 diagnoses were made in 2012 [1-3]. The development of pelvic inflammatory disease (PID) in around 10% of women with untreated chlamydia [4] may lead to long term sequelae including infertility, ectopic pregnancy, and chronic pelvic pain [5,6]. As the majority of chlamydia infections are asymptomatic [6], many infections will go undetected in the absence of screening.

Annual chlamydia screening for young adults is recommended in a number of countries; however, England is the only country currently running an organized chlamydia screening program [7], with other European countries planning screening programs in the future [8]. Australia is currently piloting an opportunistic program of chlamydia testing in general practice – the Australian Chlamydia Control Effectiveness Pilot (ACCEPt) [9]. General practice in Australia, despite being well placed to carry out screening as most young adults attend a general practitioner (GP) at least once a year, has low rates of testing at 12.1% in women and 4.8% in men [8].

As part of ACCEPt, the potential role of the practice nurse (PN) to contribute to increasing testing rates in general practice is being investigated. Recent years have seen growth in both the numbers of PNs and scope of their roles in Australia. Whilst a role in preventative and sexual health, and chlamydia testing in particular, is not as well established as in countries such as the United Kingdom, recent research suggests Australian PNs may be willing to expand into this area [10-12]. There is very little research relating to PNs’ role in chlamydia testing, but evaluations of a small pilot study of nurse-led testing in New Zealand and partner notification performed by PNs in the UK have shown that such PN-led strategies may be effective [13,14]. Research examining GPs views regarding PN involvement in sexual health is limited. Recent Australian research found a lack of support and recognition among some GPs for an expanded PN role in sexual health care [10], whilst in as yet unpublished research, PNs have reported that they were willing to have an increased role in chlamydia testing, but felt GP attitudes may be a barrier (unpublished observations -Lorch). In this, the first qualitative study to address the issue of chlamydia testing specifically, we aim to explore GPs’ opinions and attitudes towards PNs taking a role in chlamydia testing and partner notification.

## Methods

### Setting

The Australian Chlamydia Control Effectiveness Pilot (ACCEPt) is a randomised controlled trial of an organised program of chlamydia testing in general practice. It aims to determine whether annual chlamydia testing of 16–29 year old women and men can reduce the prevalence of chlamydia. A total of 134 general practice clinics in 54 rural areas of four Australian states, along with 9 in metropolitan areas were enrolled. Areas were randomised to either receive an evidence-based, multifaceted intervention developed to facilitate an increase in chlamydia testing or to continue with usual care [9]. An optional component of the intervention arm allowed the general practice clinics to involve PNs in chlamydia testing. The PNs at these general practice clinics underwent a training session and received a comprehensive chlamydia education pack. Financial incentive payments were made to the general practice clinics for chlamydia tests that involved input from a PN.

Australian general practice clinics (hereafter referred to as “clinics”) operate as small businesses. They receive the majority of their funding from the Australian government through the Medicare Benefits Schedule (MBS) on a fee-per-service basis. Until recently the sole source of funding for PNs in most clinics came from government rebates, in the form of Medicare PN “item numbers”. These item numbers covered a limited number of services provided by PNs, including wound dressings, Pap smears and immunisations. Incentive payments for the employment of PNs were also available, but only to remote, rural and some outer urban clinics [15]. In 2012, following the introduction of the Practice Nurse Incentive Program (PNIP), the incentive payments and six PN item numbers were replaced with a single funding stream for PNs. The PNIP is available to clinics in all geographic areas, and also provides additional funding to rural clinics [16].

### Study design

#### Participants

At trial baseline and prior to randomisation, in depth telephone interviews were conducted with GPs employed in participating clinics. GPs were selected using purposive sampling [17], with consideration given to geographical location, size of the clinics, age and gender of the GP to ensure variability of participants.

### Consent

GPs were contacted by telephone and invited to participate. Consent was obtained for an interview be conducted at their convenience. The GPs who were interviewed received a $100 voucher for their time.

### Interview guide

The semi structured interview guide was drafted, reviewed and refined by ACCEPt research staff. The interviews covered the broad domains of GPs’ perceived facilitators and barriers to chlamydia testing and management; the acceptability of ACCEPt to GPs; the perceived external and contextual factors affecting chlamydia testing rates and GPs’ self-reported chlamydia-related clinical practices. A number of questions related specifically to PNs’ current role in preventative and sexual health and GPs’ opinions around PN involvement in chlamydia testing and partner notification. Demographic information was also collected.

### Data collection and analysis

Interviews took place between March 2011 and July 2012, were conducted via telephone, recorded and transcribed verbatim. Data saturation was soon reached, but sampling continued to include representation of GPs from all geographic areas in ACCEPt. The range of interview length was from 20–35 minutes, with median length 30 minutes. Data relating to PN involvement in chlamydia testing and management were extracted from interview transcripts. NVivo qualitative data analysis software (QSR International Pty Ltd. Version 10, 2012) was used to organize and code the data, which was thematically analysed using a conventional content analysis approach [18]. A broad coding framework was developed initially using the main categories within the interview schedule. After multiple readings of transcripts, this framework was refined and data were grouped, labelled and emerging themes identified. Using analyst triangulation, interview transcripts were independently coded by one author, and a sub-set checked and compared by a second author to ensure consensus on emergent themes.

### Ethical approval

ACCEPt received ethical approval from the Royal Australian College of General Practitioners National Research and Evaluation Ethics Committee, the Aboriginal Health and Medical Research Council Ethics Committee and the University of Melbourne Human Research Ethics Committee.

## Results

Sixty three GPs were invited to participate and 44 agreed (70%). Of the 44 GPs interviewed, 39 were from rural areas in Victoria (15), New South Wales (11), Queensland (10), and South Australia (3), with 5 from urban areas within Victoria. Table 1 shows characteristics of participants. The themes identified during the interviews were PNs’ current role in preventative and sexual health; GPs’ support for PN involvement in chlamydia testing and management; GP’s concerns regarding PN involvement in chlamydia management, and education and training for PNs.

**Table 1:** **Participant characteristics**

**Chracteristics**	**n = 44 (%)**
**Sex**	
**Male**	27 (63)
**Female**	16 (37)
**Age group**	
**<30 years**	2 (4)
**30-44 years**	21 (48)
**45-59 years**	18 (41)
**>60 years**	3 (7)
**Location of GP**	
**Victoria – Metro**	5 (11)
**Victoria – Rural**	15 (34)
**New South Wales**	11 (25)
**Queensland**	10 (23)
**South Australia**	3 (7)
**Size of practice (Number of GPs)**	
**≤4**	25 (57)
**>4**	19 (43)
**Years in medical practice**	
**<15 years**	11 (25)
**15-29 years**	22 (50)
**30+ years**	11 (25)
**Years in general practice**	
**0-10 years**	18 (41)
**11-20 years**	13 (29)
**20-30 years**	7 (16)
**30+ years**	6 (14)
Employment status*	
**Full time**	37 (86)
**Part time**	6 (14)
**Sexual health training**	
**Yes**	9 (20)

*1 unknown.

### PNs’ current role in preventative and sexual health

Most of the GPs reported that PNs at their clinics were involved in preventative health activities such as immunisation or, most commonly, related to chronic disease management.

*We do a diabetic clinic…a lot of that would cross over into preventative health. Immunisations are preventative as well, they do adult immunisations.* GP65 (Rural).

*A lot of the chronic disease management stuff is semi-preventive in that we are trying to anticipate problems that might arise in future and manage those through things like diet and exercise, appropriate medication, and so on. They do a lot of our vaccinations particularly in flu season.* GP1 (Rural).

A number of GPs described how the PNs in their clinics held a specialised role in women’s health, and most PN involvement in sexual health occurred in the context of activities related to this role, such as cervical screening and well-woman checks.

*When it comes to sexual health it would probably be mostly limited to opportunistic prevention during Pap smears because our nurse always takes histories and she does the full deal not just the Pap smear.* GP2 (Rural).

*They mainly do women’s health checks where they do Pap smears. They certainly would offer any screening tests to any women that they thought it might benefit. And I suppose generally we have offered it to anyone under 40 but it depends on people’s lifestyle.* GP21 (Rural).

Many of the GPs felt that PNs could be utilised more effectively for preventative health activities, including sexual health, which was an area into which a few were already expanding.

*If we can broaden our preventive health campaign to things outside diabetes and ischaemic heart disease then we can incorporate a lot of other different conditions and target healthy people more than we are doing. At the moment we are mainly targeting sick people.* GP79 (Rural).

*We are just about to start a young person’s sexual health clinic. The plan is that she will see patients, take histories and do chlamydia testing. We are just about to expand her role because sexual health is something she does well.* GP18 (Rural).

### GPs’ support for PN involvement in chlamydia testing and management

All but one of the GPs expressed support for the idea of PNs becoming more involved in chlamydia testing and management. They felt that PNs were able to spend more time than GPs with patients undertaking education and follow up.

*I would love to work with a practice nurse who does all our Pap smear and chlamydia tests… I am very happy for that. I worked with nurse practitioners in England I have no objection to them running their own clinics.* GP15 (Metro).

*I think they do have time to spend with patients more than we do, and therefore they can spend more time in explaining the benefits of having something done and also have more time for education and follow-up.* GP28 (Rural).

A number of GPs also believed that within the potentially sensitive area of chlamydia management, patients would feel more comfortable and less intimidated talking to a PN.

*Patients probably don’t find nurses as imposing as speaking to their doctor…speaking to a nurse they might be a bit more comfortable.* GP34 (Rural).

*I think it is easy for a nurse to talk to a patient especially in a younger age group, to talk about such things because patients probably relate a bit more to nurses… they have a more gentle approach.* GP60 (Rural).

A few GPs also thought that PN involvement may benefit not only patients, but GPs also, offering some relief to their considerable workloads.

*That is definitely a good idea because we are under pressure for time, so basically if we are running around like always you know they could take part in screening.* GP72 (Rural).

*It is helpful for the patient and doctor as well because sometimes we are so busy. We don’t have enough time to talk preventative health. For us, junior doctors, maybe we have more time but I see the senior doctors, they don’t have enough time*. GP70 (Rural).

### GPs’ concerns regarding PN involvement in chlamydia management

Despite overwhelming support for the idea of increased PN involvement in chlamydia management, a number of concerns related to funding, PN workload and privacy/confidentiality were voiced.

### Funding and remuneration

Many of the GPs expressed concern around the funding and remuneration of PN chlamydia management. It was felt that the model of funding current at the time, rebates for specific activities performed by PNs, did not fully cover the costs of PN activities and restricted expansion of their roles.

*I think if the government was prepared to let us raise the fee for nurse consultations that reflected the time that they put in we would probably use them for more. A lot of the time at the moment they do things and we get no payment for their time.* GP29 (Rural).

*In places like the UK the nurses are quite active and they do a lot of things themselves…A lot of the things that the nurses could be doing here they don’t do because of billing… we (GPs) get more money if we do it. Definitely the nurses could play more of an active role than they are but I think the reason that that doesn’t happen so much is because of money and billing*. GP34 (Rural)

A specific item number for PN chlamydia management was suggested as a means to cover the costs of the extra resources needed for the activity, but as another GP pointed out, PN item number rebates were often insufficient.

*We could do it but then there would have to be funding to actually make sure that we have got enough nurse time available. I don’t know at this stage whether there is an item number for this. It would actually have to be an item number that made it worthwhile financially because we are entirely dependent on the income.* GP21 (Rural).

*Something like the rebate for a Pap smear does not pay for their time…The rebate from memory is about $12 to do a Pap smear and if they are doing a female health check as part of the Pap smear that is a 45 minute consultation, so they are not even paying their wage, so they can’t work independently. So we don’t do it.* GP8 (Rural).

Concerns around this area also went beyond Medicare funding and to the issue of PNs working autonomously.

*The question is should nurses be used that way? They could but it comes back down to who pays and if it is Medicare that pays then I am not so sure that Medicare is very comfortable with nurses initiating that and doctors signing off especially when the doctor is not really involved in the care of that patient.* GP26 (Metro).

### Workload and time pressures

Current workload and time pressures that PNs experienced were factors discussed by some GPs when considering how PNs could increase involvement in chlamydia management.

*We see about on an average more than 100 patients a day… we used to have another nurse come in twice a week and she would do all the other things that our main practice nurse wasn’t able to do. She has stopped coming and that has been a terrible loss for us…I think the nurses have a huge role to play and we are probably depriving the community by not having another person to do that sort of thing.* GP45 (Rural).

*Again it is time…She is doing a good job but there are huge loads. Yeah just the transfer of diabetes care into general practice has swamped our nurse. So it leaves little room for chlamydia!* GP15 (Metro).

### Privacy and confidentiality in small towns

Matters of privacy and confidentiality were brought up by a number of GPs, with those in rural areas concerned about the issues related to living in small country towns.

*Possibly, I think one of the issues in a small town like ourselves often patients are known and we would just have to be careful that wasn’t a put off for the patients. We would just have to be a little cognisant of the fact that we live in a small community.* GP29 (Rural).

These concerns were voiced in particular around PNs’ involvement in partner notification.

*I am not sure about that (partner notification). I think is a difficult thing. There are issues involved with that as far as confidentiality. If a person has only got one partner and then you ring them up and say, “You know we want to follow you up we think you both have had some chlamydia contact,” well it is pretty obvious who the person is that has been here.* GP28 (Rural).

*I think it gets a bit difficult because in a country town … one of our nurses had four children here and they went to the local high school so their children know a lot of people in town and I think it would be a bit awkward if you had to ring up someone and said, “Look you know…” And medico-legally I mean confidentiality I always sort of worryabout.* GP4 (Rural).

### Education and training for PNs

Many GPs felt that the provision of education and training, in both theory and practical skills, was an important facilitator for PN involvement in chlamydia testing and management.

*I think they should have proper knowledge of chlamydia… how can we treat it and do we have to do any follow up after? If it’s complicated chlamydia like PID, what is the management? They should know more about it so they can give proper health education to the patients.* GP20 (Rural).

*Nurses could organise urine testing or swabbing, they are very unlikely to want to organise swabbing because that requires the skills necessary for doing virtually the equivalent of a Pap smear and some nurses are comfortable with that and some are not. But look they can be trained; it is undoubtedly easy to train anyone up to that standard.* GP26 (Metro).

*I think they should be specifically trained to do that. I think if the approach is on sexually transmitted infections I think they would need to be trained for that, the way they approach them, how they go about the investigations*. GP39 (Metro).

## Discussion

The results of this first qualitative study of GP attitudes and opinions regarding a PN role in chlamydia management suggest that most GPs support PN involvement in testing and partner notification and feel PNs are well suited to the role. Despite GP support however, a number of concerns were raised, such as PNs current workload and time pressures, privacy and confidentiality issues in small towns and in particular, the funding and remuneration of PN activities in this area.

The potential contribution that PNs could make to increasing chlamydia testing rates with an expansion of their role in Australian general practice has previously been highlighted [19,20]. Within the Australian public hospital system it is being recognised that, as in the UK, appropriate utilisation of the skills of nurses and expansion of their role to undertake routine, low-risk procedures usually performed by doctors could lead to considerable financial savings and benefits for both patients and staff [21]. This can also apply within primary healthcare, where workforce shortages and the increasing burden of chronic disease are placing huge demands on GPs. These demands necessitate a broader and more flexible use of PNs’ skills if targets such as an increased focus on preventative health and an increase in opportunities for priority groups, such as young people, to access STI/chlamydia testing are to be realised [22].

The GPs in our study not only supported but recognised the possible benefits of a PN role in chlamydia management, such as a potential easing of their workload and improved patient service due to PNs better rapport with patients. However, despite the positive views expressed by our participants, recent research suggests the attitudes of some GPs towards PN involvement in sexual health may impede an extended role for PNs in chlamydia testing. Abbott et al. [10] explored the involvement of PNs in sexual health care and found that whilst some GPs saw sexual health as a central component of the PN role, others failed to recognise it as important. Furthermore, the PNs with an interest in sexual health felt constrained from utilising their skills due to lack of GP recognition and support. The issue of GP attitudes as a significant barrier to PN involvement to chlamydia testing was also raised in a series of interviews carried out with 23 PNs participating in ACCEPt (Unpublished observations – Lorch). It has been suggested by Abbott et al. that promotion of the PN role in sexual health to not only GPs and PNs, but also government, professional and educational organisations, may serve to increase this support and recognition, raise awareness and provide validation of the PN role [10].

A number of issues of concern that could act as potential barriers to PN involvement in chlamydia management were identified by the GPs. Some felt that PNs were experiencing the pressures of limited time and increased workload that GPs themselves were facing. This is a barrier consistently raised to chlamydia testing for GPs both in Australia and the UK [23], and for Australian PNs themselves [12], but it is not insurmountable. The provision of education and training for PNs, an important component of the ACCEPt PN intervention and raised as a facilitator by our GPs, can incorporate examples of approaches to testing that PNs can implement in their clinics to minimise time needed to undertake chlamydia testing consultations. These approaches may involve the implementation of “chlamydia testing pathways” aimed at streamlining and simplifying the testing process and also the use of predetermined “scripts” when offering and undertaking chlamydia testing with patients.

A number of the GPs in clinics in small rural towns raised concerns about privacy and confidentiality, particularly if PNs’ were involved in partner notification. They felt that young patients may be “put off” if PNs and their families were known to them. A similar concern was also raised by PNs participating in ACCEPt (unpublished observations - Lorch). Past research has demonstrated that young people in small towns have the same concerns [24,25]. However, results from a recent cross- sectional chlamydia prevalence survey undertaken in mostly rural Australian towns suggest that whilst young people may have concerns around initiating testing, most find it acceptable to be offered a test, with 70% of the 16–29 years olds approached during the survey agreeing to chlamydia testing and 86% of these reporting that they were attending their local clinic [26].

The issue of remuneration and funding of PN involvement in chlamydia testing, however, was of particular concern to the GPs. The majority of the interviews were undertaken whilst the previous fee-for-service PN funding model was still in operation. This model of funding, as highlighted in the interviews, had drawbacks for both the PN and the GP. The range of PN specific activities that attracted a rebate was very narrow and in some cases restricted the PN role to tasks that generated revenue for the clinic. However, PNs also engaged in a wide range of other activities that did not attract a rebate and were therefore unfunded [27,28]. A specific PN chlamydia testing and management “item number”, which did not exist at the time of the interviews, was suggested by some GPs as a facilitator. This could not only ensure the cost of PN time was funded, but also enable PNs to be autonomously involved in the process of chlamydia testing and management. Funding for PN involvement in a “young person’s” preventative health check, which could include screening for chlamydia/STIs and other psychosocial risk behaviours, is another possibility. Recent research has shown that general health checks among adults are not effective in achieving improved patient health outcomes [29]; however, evidence of the impact of preventative health screening of young people, and the role of the PN in this area, is insufficient. The prevention access and risk taking in young people (PARTY) project is an Australian cluster RCT which is currently evaluating an opportunistic preventative screening and brief counselling intervention aimed at young people in general practice, involving both GPs and PNs [30]. Early results suggest that with appropriate training, and in collaboration with GPs, PNs are able to conduct these preventive health checks for young people and find this role acceptable [31,32].

The introduction of the new model of PN funding, the PNIP, may also address the issue of funding for PN involvement in chlamydia testing and management. The replacement of a number of PN item numbers with a single funding stream is a change which aims to support an extended and enhanced role for PNs, enabling flexibility for them to undertake a broader range of activities (including prevention) based on patient and clinic need rather than income generation. With no restrictions on activities covered by funding, the PNIP may provide the opportunity for PNs to be increasingly involved in chlamydia testing and management, freeing up time for GPs to deal with more complex cases and increasing access to testing for young people [33]. However, whilst implementation of the funding model is still in the early stages and its impact on PN roles is yet to be formally evaluated, it has been suggested that the PNIP may be seen by GPs and practice managers as limiting the work PNs can perform since there are no longer financial incentives attached to “specific” activities [34]. Further work may be needed to inform and support GPs and practice managers in understanding how the scheme can work to the advantage of not only themselves, but to PNs and most importantly patients.

The strengths of this study include the recruitment of GPs from a diverse range of clinics and locations across Australia and a response rate of 70%. There are also some limitations. The GPs who agreed to be interviewed may represent a biased sample of GPs with a special “interest” in sexual health, and this bias may account for the overwhelming support of PN involvement in chlamydia testing that the GPs in our study demonstrated. Furthermore, the sample was drawn from GPs participating in ACCEPt who were from mostly rural and regional areas. Abbott et al. found that PNs working in rural vs metro settings had a stronger role in sexual health, and were more supported in this role by GPs [10]. Although the views of GPs from metro and rural settings were for the most part similar, the issues unique to rural general practice, such as ongoing workforce shortages and higher rates of chronic disease than in metro areas [35,36] mean that our study may not represent the views of all GPs across Australia. Finally, as with most qualitative research, the results are based on a relatively small sample size. However, the purpose of qualitative research is to gain either breadth or depth of opinion, in contrast to the purpose of quantitative research which aims to describe the frequency of such views. This study had a sample size of 44, which is high in terms of qualitative research. In addition, a broad range of GPs was included in our sample, but despite this, data saturation (the point at which no new data emerged) was reached early on in the interviewing process, suggesting that their views were fairly consistent.

## Conclusion

In conclusion, untreated chlamydia infection may lead to serious reproductive health issues for young people and is thus a significant public health concern. The impact of increasing chlamydia testing rates in general practice on the burden of the disease in this population is currently being investigated in Australia, with the role of the PN being investigated for the first time. Our study suggests that whilst some GPs recognise and support the potentially valuable contribution that PNs could make, barriers to PN involvement in this area may impede progress. Further research into the effectiveness and feasibility of PN chlamydia testing is warranted, and in particular, the mechanism of funding for a PN role in chlamydia testing and other areas of expanded practice requires further consideration.
